# A review of drug knowledge discovery using BioNLP and tensor or matrix decomposition

**DOI:** 10.5808/GI.2019.17.2.e18

**Published:** 2019-06-27

**Authors:** Mina Gachloo, Yuxing Wang, Jingbo Xia

**Affiliations:** Hubei Key Laboratory of Agricultural Bioinformatics, College of Informatics, Huazhong Agricultural University, Wuhan 430070, China

**Keywords:** BioNLP, drug knowledge discovery, tensor decomposition

## Abstract

Prediction of the relations among drug and other molecular or social entities is the main knowledge discovery pattern for the purpose of drug-related knowledge discovery. Computational approaches have combined the information from different resources and levels for drug-related knowledge discovery, which provides a sophisticated comprehension of the relationship among drugs, targets, diseases, and targeted genes, at the molecular level, or relationships among drugs, usage, side effect, safety, and user preference, at a social level. In this research, previous work from the BioNLP community and matrix or tensor decomposition was reviewed, compared, and concluded, and eventually, the BioNLP open-shared task was introduced as a promising case study representing this area.

## Background of Drug-Related Knowledge Discovery

Drug-related knowledge discovery is the process of discovering novel drug targets, drug-side effects, drug-drug interactions (DDIs), drug-disease or drug-indications interactions. The novel knowledge discovery has mainly led to better understanding of the molecular bases of drug efficacy, and with focus on the application scenario of new drug discovery, drug development or drug repurposing [1], i.e., search and replacement of compounds developed for specific diseases [2]. Drug discovery is usually initiated by an experimental method or computational method. Experimental methods, either *in vivo* or *in vitro*, are more acceptable by the clinical community. However, the disadvantages of these methods, such as cost and time-consumption, are also obvious. The computational way, also known as *in silico* method, is mainly to perform the knowledge discovery under data mining instead of experimental (“wet lab”) manipulations. Early in 2009, a review in *Nature* [3] claimed that *in silico* predictions for drug discovery has come of age, and so far, PubMed has collected over 41 thousand papers about *in silico* drug knowledge discovery.

## *In Silico* Methods for Drug Knowledge Discovery

Generally, *in silico* methods are a computational way to perform knowledge inference by using data mining, with less time-consumption and including machine learning, molecular docking, pharmacophore structure, structure-activity relationships (SAR), quantitative structure-activity relationship (QSAR), and combination methods. Drug-knowledge discovery with *in silico* methods mainly identified core molecular entities, including genes, proteins, therapeutic compounds, and other “omics” information and henceforth, to explore the novel link between them [4]. Though chemoinformatics methods, such as SAR or QSAR, have made great success in screening chemical libraries, the huge body of candidate chemical compounds has led to overload calculation and made these methods far from perfect [5]. Thanks to the rapid emergence of deep neural network since the early 2010s, deep learning strategies have undoubtedly manifested their computational advantage over chemoinformatics strategies for drug screening [6], and made it another application field of deep neural networks. In the meantime, chemoinformatics strategies mainly focused on novel drug-target identification or DDIs prediction, instead of drug-side effects or drug-disease pairs. With higher odds of success, knowledge discovery tasks of recent ones relied heavily on structured knowledge entires came from bioinformatics-based data base searching or natural language processing (NLP)–aided automatical curation.

In this review, we mainly focus on two typical *in silico* methods of drug-related knowledge discovery. One method is text mining, i.e., Biomedical Natural Language Processing (BioNLP). Another one is a knowledge discovery method, with low rank approximation of drug data with a form of tensors or matrices. As structured knowledge entries were supportive to resolve most drug-related knowledge discovery tasks, NLP methods are regarded as a good addition to traditional *in silico* methods. In addition, the popularization of knowledge graphs, in recent years, has dramatically encouraged the promising application of knowledge inference in drug-related knowledge discovery. Tensors or matrices were treated as natural data structures, to contain drug knowledge entries, and tensor or matrix decomposition served as a rough approximation of novel link discovery.

### Text resources and BioNLP methods for drug-related knowledge discovery

BioNLP is the application of NLP methods to biomedical entities such as macromolecules and relation extraction between protein-protein or drug-drug interactions. As a hyponym word for NLP, the definition of BioNLP appeared in the early 1990s [7], when distributed word representations and applications in BioNLP were introduced. With the fast accumulation of written material of scholarly publications and clinical narratives, the BioNLP community, formed in the late 1990s and various named entity recognition (NER) tools were developed for the purpose of biomedical applications such as DDIs, data base curation, ontology design, and so on [1].

In this section, we review the development of BioNLP in drug-related knowledge discovery by categorizing the resources for which type of research was performed. Three kinds of text resources, i.e., large-scale curation data, small-scale corpora, and heterogeneous data, were introduced, as well as drug-related discovery research approaches based on them. Here, PubMed and OMIM were introduced as two representatives of large-scale curated data, which as a tradition served for drug-related knowledge discovery for years; corpora emerged from small-scale data aiming for serving high quality text mining upon large text data; and finally, multi-omics data was introduced as heterogeneous data.

#### Large scale curation data and drug-knowledge discovery in a wide range

Released for the first time in 1996, PubMed has long been the main text resources for the BioNLP community to collect references and abstracts on life sciences and biomedical topics [8].

The 2014 version of PubMed Medline was explored by Yang et al. [9] through lexicon filtering and dependency parsing tree establishment. They used trigger word learning to extract relationships between diseases-genes and genes-drugs, After obtaining 114,381 disease-gene and 176,219 gene-drug link pairs, an ABC model was applied to extract the indirect link between disease and gene by considering disease-gene as A-B and gene-drug as B-C.

NER tools were developed, as well, among the BioNLP community, among dozens of popularized NER tools, including tmChem [10], DNorm [11], GNormPlus [12], and tmVar [13]. These were regarded as successful representative tools for recognizing chemicals, diseases, genes, and variations.

In the meantime, emergence of deep learning strategies in NLP propelled bio-NER dramatically, by introducing novel and sophisticated deep neural network training models, in the manner of classifier and word embedding. First, deep learning brought a new generation of neural networks as an effective classifier, i.e., long short-term memory (LSTM) neural networks; Second, deep learning introduced semantics consideration, like word embedding, as input, and enhanced the NER algorithms. For example, Habibi et al.’s work [14] was typical, which fully made use of CRF, LSTM, and word embedding, to extract entities including drugs from text, and the results of this work indicated that deep-learning methods performed better than other biomedical NER methods. The attempts of BioNLP community made the massive bioentity information retrieval more accessible.

As a user-friendly platform run by NCBI, PubTator [15] timely offered the PubMed-scale NER service to tag the above entities. By integrating the tagged entities of PubTator into the Stanford parsing tree, Percha and Altman [16] grouped PubMed sentences into semantically-related categories, to provide relations between entities and pairs, for each sentence. For instance, six groups of gene-chemical pairs were carefully defined in this work, i.e., drug target, metabolism, transport, inhibition, agonism, and antagonism. Finally, sophisticated semantic relations were mined out, such as DDIs, and variations in drug responses.

Except PubMed, there were several text resources serving for drug-related knowledge discovery. Online Mendelian Inheritance in Man (OMIM, https://www.omim.org/) [17] for drug mechanism, and ClinicalTrails.gov (https://www.clinicaltrials.gov/) for drug usage.

OMIM, a popular knowledge base of human genes and genetic disorders, offers enriched text sets for addressing phenotypes of mutated genes. Wang and Zhang [18] manually curated the functional change mutations type, i.e., loss of function (LOF) and gain of function (GOF) mutations. It was stated that LOF and GOF recognition worked for novel drug discovery, in terms of core gene function change. Wang and Zhang [18] hypothesized that the “antagonist” chemical maps to a targeted gene with GOF, while another “agonist” chemical mapped to the gene with LOF. This hypothesis offered a straightforward rule for gene-drug pair filtering. Zhang et al. [19] employed OMIM and PubMed to gather GOF and LOF knowledge on the pathogenesis of antidiabetic targets, finding nine drugs for treating diabetes.

Besides PubMed text resources for published papers, and OMIM for curated heredity-centric knowledge text, ClinicalTrails.gov is a representative of an electronic health record (EHR) text resource, which was established in 1999 [20]. ClinicalTrails.gov contains various information about medical clinical studies in humans, and the open access policy made it widely used. For example, Su and Sanger [21] extracted serious adverse events SAEs data from the text in ClinicalTrials.gov, and ranked drugs by SAEs data, to find those with the least SAE. Then, new drugs could be predicted according to their SAEs. For example, Xu et al. [22] extracted gene alterations and identified cancer treatment trials by developing a semi-automatic framework on documents at CliniclaTrails.gov. In this research, they used three steps including: collect candidate trials about cancer treatment trials, score each candidate trials, and manually review trials with lower scores.

EHR data is a popular source information of clinical and transnational research for drug repurposing. Banda et al. [23] used four sources information from EHRs including public database, source of spontaneous reports, literature and non-EHRs DDIs predication methods to prioritize drug- drug-event association. It should be noted that the abundant clinical information in EHR data made it possible to serve for various precisional medical discovery. Denny et al.’s PheWAS [24] combined long temporal scale EHR data with genomics variation information, and proposed phenome-wide association study to trace core single nucleotide polymorphisms and disease trajectory. The emerging cross disciplinary research based on EHR as well propelled the research issues from Medical Natural Language Processing (MedNLP) [25,26].

In all, the development of large data resource knowledge discovery unveiled the following tendencies:

(1) PubMed is still the main open access resource for large scale resource, meanwhile, lack of other text resources with comparable level and restriction of full text access hinder the development of large scale knowledge discovery for bio-text miners.

(2) After years of development, NER of biomedical entities is not technical headache any longer, and make it possible to run comprehensive knowledge extraction tasks.

(3) As a result, a combination of full open access to PubMed-wide knowledge discovery and restricted access EHR data access for drug knowledge is a main research pat- tern in the next decade.

#### Corpora and purposes for drug-related text mining

Early attempts to apply BioNLP to knowledge discovery was propelled by the benchmark NLP dataset corpus. A well-structured corpus experiences a rigid evaluation procedure that ensures its usability. The steps included annotation guidelines design, annotation testing, and inter-annotator agreement computation.

The pioneer work was the corpus used in DDIs of DDI 2011 [27], DDI 2013 extraction challenge [28], and SemEval 2013 task 9 [29]. In early attempts, Segura-Bedmar et al. [27] used POS-tagging, lemmatization, and chunking as features of a shallow linguistic kernel method, to perform DDI extraction. To that end, Bui et al. [30] was among dozens of researchers that attended the DDI challenge, which manually created 292 relevant trigger words, converted sentences into semantic structures, extracted and fed features into a known classifier support vector machine (SVM) for DDI extraction. Afterward, Kim et al. [31] used SVMs, as well as performing DDI 2013 challenge, but with richly combined features, including word features, word pair features, dependency graph features, and parse tree features.

Corpora design, and its applications, gradually played substantial roles in drug-related knowledge discovery. In 2016, for the purpose of oncology knowledge discovery, Lee et al. [32] created a cancer and antitumor Biomedical entity Relation ONcology COrpus (BRONCO), which focused on the variant-centric entities including genes, diseases, drugs, and cell lines. Although BRONCO was a disease-oriented corpus, it focused on drugs, and Lee et al. [33] used this corpus to evaluate and develop a mutation-gene-drug discovery pipeline.

Focus on adverse reactions (ADRs) or side effects on drugs has attracted the attention of corpus designers. In that regard, Fang et al. [34] illustrated proper terminology discrimination upon ADR corpus design. A recent ADR-oriented corpus was created by an NCBI team Demner-Fushman et al. [35], i.e., Text Analysis Conference (TAC) 2017 drug labels corpus, which annotated labels of two hundred Food and Drug Administration approved drugs. The mentioned topics they annotated covered “severity,” “drug class,” “adverse reaction,” etc., which were fairly usable for ADR evaluation of drugs. ADR extraction was among the successfully held tasks of TAC 2017 and 2018 [36], and afterwards the same NCBI team constructed MEDIQA challenge, an Association for Computational Linguistics–community challenge for the question entailment of medical records [37] which expanded the drug-related ADR extraction to wider clinical scenarios, also known as MedNLP.

Another focus on drug-related corpus construction is on drug repurposing. Until now, the corpus working on drug repurposing was rare. Recent progress came from Wang et al.’s work [38], which designed an “active gene annotation corpus (AGAC)” to cultivate functional change of mutated genes. AGAC aimed to capture LOF- or GOF-mutated genes, and made it possible to find “agonist vs. LOF” and “antagonist vs. GOF” pairs for “drug vs. gene.” This was a nice addition to a mutation-centric corpus for the purpose of drug repurposing [38].

The development of drug-oriented corpora design showed clear tendency as below.

(1) DDIs were a key focus in corpora design, and the DDI corpus has long been a tradition in drug-related corpus construction.

(2) Disease-oriented corpora covered drug- related knowledge curation, which served directly to specific disease and focused on tumors as targets.

(3) Drug-related ADR or side effect information was a focus in corpora design which served for drug effect, and as well led to expanded attention in medical and clinical applications.

(4) Mutation-centric corpus was a novel addition to the drug-related corpora, which was aimed to the application of drug repurposing.

#### Heterogeneous data for drug-related knowledge discovery

Unlike traditional text data, heterogeneous data is generally non-scientific text, like so cial media and various omics data, including genomic or proteomic data. While the non-scientific text enhanced research studies, with social concerns such as drug abuse, drug misuse, and drug safety, the various omics data achieved success under the collaboration of BioNLP and bioinformatics community.

Just like Twitter served well for drug prescription and drug abuse [39], social media allowed fast tracking of public opinion, and became popular resources for adverse drug reaction mining [40,41], drug misuse [42], drug safety [43], etc. It was worth noting that social media texts were mainly integrated into research with social issue topics, instead of drug knowledge in the molecular level.

With emergence of multi-omics data, the integration of text data with genome, or protome data attracted attention from a cross disciplinary view, for the purpose of drug-gene linking discovery. Early attempts of linking chemical to candidate genes was performed in late 2000s by Li et al. [44], who showed a significant combination of traditional bioinformatics and BioNLP approaches. This study used Online Predicted Human Interaction Database (OPHID), a predicted protein association network database, to obtain protein networks of Alzheimer disease, retrieved from disease-drug-protein links from PubMed, and formed a reliable connectivity map.

In most cases, multi-omics data integration led to indirect link discovery between drugs and their targeted proteins or candidate loci. Zhang et al. [45] obtained a colorectal cancer-related gene list by text mining from PubMed and then integrated genomics data and proteomics data to identify the more risky loci associated with colorectal cancer. Barupal et al. [46] investigated metabolic genes as therapeutic targets in breast tumors by using multi-omics data and text mining. Meanwhile, Long et al. [47] identified and validated oncogenic biomarkers of pancreatic cancer, through integrative text mining and omics-based translational modelling. Such progress also reflects the mainstream data fusion research idea within the bioinformatics community.

To conclude, the availability of the heterogeneous data propelled drug-related knowledge discovery both in social and bioinformatics domains.

(1) Social media data became an exclusively important resources for collecting public opinion, helping to resolve several drug-related topics, such as drug safety, drug usage, or drug side effects.

(2) Integration of text data with multi-omics data became a tendency upon drug-gene linking or therapeutic target discovery, and huge text data was regarded as one member of omics data from the view of the bioinformatics community.

### Matrix or tensor decomposition methods for drug-related knowledge discovery

Matrix factorization or decomposition are important techniques for extracting information from a matrix or a tensor [48]. Basically, a matrix A∈ R*^m×n^* refers to a *m × n* data array, which is suitable for storing and linking two entities. Meanwhile, an *M*-way tensor R*^n1 ×n2 ×···×nM^* provides a higher ordered structure, which is capable of storing *M* different kinds of entities. The computational decomposition (sometimes called factorization) result of a matrix or a tensor led to a so-called low rank approximation of the original structure, and made a basis for novel link discovery.

If compared with great amount and various patterns of BioNLP research on drug-related knowledge discovery, the research of matrix or tensor decomposition was comparatively less, and more topic-specific. In general, the adaptable data structure made it possible to illustrate higher order links, while the lower rank approximation made it a suitable one for novel link discovery. A comprehensive review of mathematical illustration of the matrix decomposition (“also known as matrix factorization”) by Wang and Zhang’s work [49] listed basic notations, definitions and detailed ideas, while Kolda and Bader [50] provided another one for tensor decomposition, including the classic CANDE-COMP/PARAFAC (CP) and Tucker decomposition. To trace the rapid development of knowledge inference in the years of knowledge graph, Nickel et al.’s RESCAL algorithm [51] made good use of tensor structure for triple knowledge. In addition, Nimishakavi’s series work on higher order relation schema [52,53], and side information integration, were representative issues in higher order link discovery and multi-resource data fusion. Since this review is mainly for concluding research on drug-related knowledge discovery, the following section reviews the matrix and tensor level decomposition and applications on drug, respectively.

#### Matrix decomposition method

Matrix decomposition obtains a sum of lower-rank matrices, and then models a small number of factors [54]. A matrix decomposition models known associations, which predicts novel drug indications.

In 2013, Zheng et al. [55] predicted new drug-target interactions by using collaborative matrix factorization. In their work, three different datasets, such as drug-target interactions, DDIs, and target-target interactions were input to build three matrices. After matrix factorization, two low-rank matrices were obtained, which approximate to the known drug-target interaction matrix, and novel relationship predictions were able to perform by the new number in the approximate matrix. In this research, three kinds of data such as drug-target interaction, drug similarity, and target similarity were input into the three matrices separately.

Similarly, Liu et al.’s work [56] presented an integrated framework to create new therapeutic associations between drug-drug, drug-disease, and disease-disease by matrix decomposition. Zhang et al. [57] proposed two projections including low-dimensional drug projection and disease projection matrix, and utilized them to factorize the drug-disease matrix. Dai et al. [58] used three interaction data, including drug-disease, disease-gene, and drug-gene interactions, to predict drug-disease association. In their work, they clustered genes by using gene-drug interactions and gene-disease interactions, respectively, and two different clustering results consisted of two axes of matrix. After matrix factorization, the novel relations between the clusters were predicted. Through tracing backing to the corresponding disease and drug of the clusters, newly drug-disease relations were obtained.

##### Tensor decomposition method

Tensor decomposition appeared early in 1927 [59] and emerged into computer science applications in the 2000s. Tensors incorporate a multidimensional array of numerical data and are applied to various machine-learning tasks [48]. Similar to matrix factorization, tensor decomposition extracted a low rank approximation of drug data, while withholding more complex data structure. To that end, Ho et al. [60] utilized tensor decomposition, in an unsupervised manner, for EHR data, and extracted candidate phenotype generation through checking interactions of diagnoses and drugs among patients. Arany et al. [61] similarly used tensor decomposition to infer drug-protein interaction types: competitive or non-competitive. This was a novel idea in this research to design a 3-way tensor with cell *ijk* represented inhibition of the *j*-th protein with *i*-th drug for the *k*-th given inhibition measure, and to decompose the tensor by using side information of chemical features.

Basically, it was a natural idea to incorporate various drug-related information into the axes of a tensor, and achieve an imaginary knowledge structure. Khan et al. [62] proposed structural toxicogenomics complex tensors by creating structure matrices with drugs and structural descriptors, respectively, a gene tensor for diseases, and post-treatment gene expression, and a toxicity tensor with drug toxicity measurements. Decomposition of the complex tensors led to predictions of toxicity of unseen drugs. Afterward, Taguchi et al. [4] performed a series of studies on identified candidate drugs, especially drugs for heart failure [63], by integrating gene expression data into a tensor decomposition model. Unlike linking drug-protein, drug-toxicity, or drug-disease pairs, linking drug-consumer led to different applications, like drug recommendations. Wang et al. [64] likewise designed a 3-way tensor with “user,” “drug” and “label,” and constructed a precise drug recommendation model.

The above methods mainly fulfilled tensor axes with various drug-related domain data like gene expression or chemical features, and then a novel link discovery was mined out from the decomposed tensor. Meanwhile, a hybrid strategy of BioNLP and tensor decompostion came from Zhou et al. [65], who used AGAC corpus [38] as a training set to perform OMIM-wide text mining, and predict novel higher order links among five entities, including genes, mutations, functions, diseases, and functional changes. In this work, new nonzero cells in the decomposed tensors were treated as novel links, among five entities, and infer the functional change of a mutated gene. Finally, agonist/antagonist drug information was extracted from DrugBank [66], and applied to help linking “agonist vs. LOF” and “antagonist vs. GOF” pairs, for the purpose of drug repurposing.

#### Research pattern of novel drug-related knowledge link discovery in the form of matrix or tensor decomposition

Among the above research studies, the characteristics of matrix or tensor decomposition method enabled investigators to input multiple data, and thus provide more comprehensive information for prediction, which may elevate knowledge prediction accuracy. The research tendency of matrix or tensor decomposition on drug-related knowledge discovery is listed below.

**Fig. 1.**
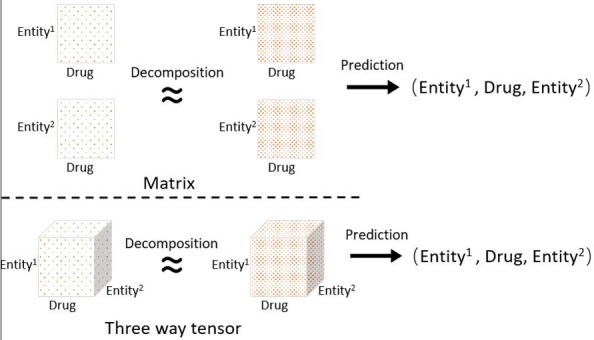
Structure of a matrix and a three way tensor.

(1) Matrix or Tensors are natural data structures to contain multiple arrays of drug-related entries. Paired knowledge entries are mapped into a matrix element, such as a drug-target, drug-drug pair, while three linked entities are mapped into a cell in tensor, such as “drug”, “user,” and “label,” in drug recommendations. Furthermore, higher order links are mapped into higher order tensors.

(2) Generally, novel link discovery is inferred from the novel nonzero cells in the decomposed matrix or tensor. Methods differ according to the chosen decomposition algorithm. For example, a new link is inferred from a core tensor after decomposition in a RESCAL-based tensor decomposition, while a nonzero cell in the approximated tensor counts as a novel link in a CP decomposition.

(3) Three way tensors were the most popular choice in the knowledge inference applications. As shown in Fig. 1, a 3-way tensor is favored more in a triple data structure than that in two matrices, thus making it convenient for high-order link data representation. It is straightforward to claim that a M-way tensor can provide a natural data structure to store higher-order links mentioning M entities. However, the higher the reach of the level, the more sparse the tensor is. This creates a computational bottleneck.

(4) Knowledge inference algorithms such as jointly decomposed matrices and tensors, bring the data fusion idea into the matrix or tensor decomposition strategy, and make it possible to perform a drug-related knowledge discovery, by incorporating various kinds of heterogeneous data.

## Conclusion

### Trends in BioNLP and drug-related knowledge discovery

The goal of drug-related discovery is to find novel knowledge for extracting drugs, and use the newly identified drugs for disease therapy. In this review, we focused on BioNLP and tensor or matrix decomposition methods to predict novel alternative therapeutic symptoms.

Recent progress in drug-related knowledge discovery led to a couple of research trends:

(1) Well-annotated corpora are a core gold standard dataset. Annotation corpora are crucial to BioNLP, and could help to retrieve and extract information from biomedical text, and also provide standard data for repeatable training and evaluation of BioNLP.

(2) NER tasks are replaced by more complicated knowledge curation tasks, in the BioNLP community. Information from text can be extracted by BioNLP, which could be the original data to find novel knowledge through prediction models. With the recent development of PubTator, NER, and term normalization, are properly solved, while aiming to curate all of PubMed.

(3) The application of BioNLP in drug-related knowledge discovery requires deepened integration of multi-omics data. Cross-disciplinary collaboration among BioNLP, MedNLP, and bioinformatics communities is a promising approach.

(4) Knowledge inference, based on tensor or matrix decomposition, is regarded as a reliable prediction model. The integration of algorithms and theorems, developed in knowledge graphs, is a promising approach to resolve various drug-related knowledge discoveries.

### BioNLP Open Shared Task: AGAC track

To encourage cross-disciplinary collaboration from various drug-related knowledge discoveries, shared tasks have long been a stage to gather researchers with different backgrounds, e.g., the series of BioNLP Shared Task (BioNLP-ST) workshops [67-71].

Aiming to gather text mining approaches among the BioNLP community to propel drug-oriented knowledge discovery, BioNLP Open Shared Task workshop (https://2019.bionlp-ost.org/tasks) proposed five sub-tasks (tracks). Among the five tasks, we propose a AGAC track (https://sites.google.com/view/bionlp-ost19-agac-track), for the goal of drug repurposing.

AGAC track provides an AGAC and aims to extract mutation-disease knowledge from PubMed. The mutation-disease knowledge in this track links gene-mutation-function change to disease, which not only contains the relationship between mutation and disease, but also indicates the functional change of the mutation, i.e., GOF or LOF. One application of this track is to elevate the efficiency of drug discovery, since matching drugs with their target mutated genes must consider the corresponding of the function change of mutated gene and the pharmacological activities of drugs.

AGAC track contains three different tasks.

(1) Trigger words NER: This task requires participants to recognize trigger words from PubMed abstracts, and annotate them with their corresponding AGAC labels or entities (Var, MPA, Interaction, Pathway, CPA, Reg, PosReg, NegReg, Disease, Gene, Protein, and Enzyme).

(2) Themetic roles identiftcation: Identification of AGAC themetic roles (e.g., Theme Of, Cause Of), between trigger words.

(3) Gene-function mutation-disease link discovery: Extract the gene-(mutation)-function change-biology function or disease link. For example, “Mutations in SHP-2 phosphatase that cause hyperactivation of its catalytic activity have been identified in human leukemias, particularly juvenile myelomonocytic leukemia.” From this sentence, the participants need to extract (SHP-2–GOF–juvenile myelomonocytic leukemia).

The baseline methods for task 1 or 2 was performed in Zhou et al.’s work [65], while the “agonist vs. LOF” and “antagonist vs. GOF” hypothesis for the support of drug repurposing was proposed in Wang and Zhang’s work [18]. The development of the AGAC corpus [38] laid the basis for the data availability, while PubAnnotation [72] served as the evaluation platform.
